# Host Proteins in *Echinococcus multilocularis* Metacestodes

**DOI:** 10.3390/ijms26073266

**Published:** 2025-04-01

**Authors:** Joachim Müller, Beatrice Zumkehr, Manfred Heller, Anne-Christine Uldry, Sophie Braga-Lagache, Britta Lundström-Stadelmann

**Affiliations:** 1 Institute of Parasitology, Department of Infectious Diseases and Pathobiology, Vetsuisse Faculty, University of Bern, 3001 Bern, Switzerland; joachim.mueller@unibe.ch (J.M.);; 2 Proteomics and Mass Spectrometry Core Facility (PMSCF), Department for BioMedical Research (DBMR), University of Bern, 3008 Bern, Switzerland; manfred.heller@unibe.ch (M.H.); anne-christine.uldry@unibe.ch (A.-C.U.); pmscf.dmbr@unibe.ch (S.B.-L.); 3 Multidisciplinary Center for Infectious Diseases, University of Bern, 3012 Bern, Switzerland

**Keywords:** homeostasis, helminth proteomics, host-parasite interaction, model system, systems biology

## Abstract

Metacestodes of *Echinococcus multilocularis* are the causative agents of alveolar echinococcosis, a neglected, life-threatening, zoonotic disease. To study these metacestodes in vitro, a model system using a culture medium conditioned by rat hepatoma cells is available. A key question is how the parasite interacts with the host and, in particular, which host-derived compounds are taken up. In this study, we focus on the uptake of host-derived proteins. Studies with artificially labeled proteins suggest that this uptake may occur independently of protein size or charge. Closer investigation using proteomics draws, however, a different picture. Of 1170 host (i.e., rat or bovine) proteins as identified by LC-MS/MS-based proteomics present in the culture medium, only 225 are found in metacestode vesicle tissue or fluid. Moreover, their relative abundances differ. Serum albumin, the most abundant culture medium host protein, is only the third most abundant protein in vesicle fluid, where Alpha-2-HS-glycoprotein becomes the most abundant protein. In vesicle fluid obtained ex vivo from experimentally infected mice, the situation is again different, with histone isoforms as the most abundant proteins. This suggests that while maintaining their internal milieu constant, metacestodes may adjust the spectrum of host proteins taken up. Potential uptake mechanisms and functions are discussed.

## 1. Introduction

The closely related zoonotic cestodes *Echinococcus multilocularis* (fox tapeworm) and *E. granulosus* (dog tapeworm) cause the neglected diseases alveolar echinococcosis (AE) and cystic echinococcosis (CE), respectively, in both humans and animals. In Europe, AE is the most significant foodborne parasite in humans and ranks among the top three globally [1], with an annual burden exceeding 688,000 Disability Adjusted Life Years (DALYs) [2]. CE, while ranking fourth in Europe, is the second most impactful globally, with 184,000 DALYs. Though relatively rare (~18,250 new human cases annually [3]), AE incidence is rising [4,5]; CE affects over 188,000 people each year [2] and poses a major economic burden on global livestock industries, causing losses exceeding US$ 2 billion annually [6]. *E. multilocularis* follows a predator-prey transmission cycle involving carnivores as definitive hosts and rodents as intermediate hosts, whereas the *E. granulosus* species complex primarily involves larger herbivorous mammals as intermediate host species, such as bovines or sheep [7]. Accidental hosts of *E. multilocularis* include humans, monkeys, and dogs [8]. The parasite’s metacestode stage develops in the liver, causing cancer-like growth and metastasis [9]. Without treatment, AE is fatal in >90% of cases within 10–15 years [9]. Curative treatment requires radical metacestode removal and drug therapy [9], but surgery is only viable in 20–50% of cases [10,11]. If surgery is not an option, patients receive lifelong benzimidazoles [11,12], which halt parasite growth but are not parasiticidal; thus, massive doses have to be administered throughout life [9].

The fluid-filled *E. multilocularis* metacestodes are vesicular structures comprised of an acellular laminated layer, the tegument, and the inner germinal layer, collectively called vesicle tissue (VT). The metacestodes of *E. granulosus* follow a similar structure, yet they present rather as unilocular cysts. The outer laminated layer of *Echinococcus* metacestodes is a unique carbohydrate-rich extracellular matrix covering the surface [13]. The syncytial tegument is anchored to the interior surface of the laminated layer, and the germinal layer is composed of connective tissue, muscle cells, nerve cells, glycogen/lipid storage cells, subtegumentary cytons, and undifferentiated stem cells [14]. The vesicle fluid (VF) inside the metacestodes plays a role in nutrition and the exchange of metabolites between the parasite and its surroundings. Stem cells are the only cells of the metacestode with unlimited proliferative potential, and drugs need to destroy the stem cells to achieve parasiticidal activity [15].

Larval stages of *Echinococcus* spp. and of other cestodes evolve in an environment characterized by the presence of host micro- and macrometabolites, including proteins. To what extent are host proteins taken up into these stages, and what may be their role? Early investigations have revealed that isolated *E. granulosus* metacestodes and *Taenia* sp. larvae take up the I^125^-labelled immunoglobulins [16]. Furthermore, native human serum albumin and IgG were found in *E. granulosus* hydatid cyst fluid from a patient [17]. Finally, proteomic analyses of cysts and adult stages from intermediate and definitive hosts have revealed numerous host proteins within cyst fluid and other compartments of the parasite [18,19,20]. The readout of studies with *E. granulosus* is, however, hampered by the fact that they are based on ex vivo, thus patient-borne material, since standardized in vitro culture systems for these parasites have been only recently developed [21].

The situation with *E. multilocularis* metacestodes is different. Using a culture medium preconditioned with rat hepatoma cells, they can be cultivated in vitro for several months and, therefore, provide a suitable standardized system to study host-parasite interactions [22]. A proteomic study using this system has led to the identification of 54 culture medium, thus, host-based proteins in *E. multilocularis* VF [23]. Employing the same culture system, we have succeeded in characterizing the metabolomes of metacestode VF and of metacestode culture medium [24]. Moreover, we have characterized the *E. multilocularis* proteomes in metacestode VT, VF, and in culture medium with a special emphasis on secreted antigen B isoforms [25]. In the present study, we shed light on the proteins of host origin within *E. multilocularis* VT and VF, asking the question of whether host proteins in VT and VF correspond qualitatively and quantitatively to the respective levels in the medium. Our null hypothesis is that there is no selectivity, resulting in an equal distribution of host proteins between medium, VT, and VF. Moreover, we compare the results concerning the VF proteome obtained in vitro to the proteome of VF collected ex vivo from infected mice.

## 2. Results

### 2.1. The Overall Protein Concentration in Metacestode Vesicles Remains Constant

**Figure 1:**
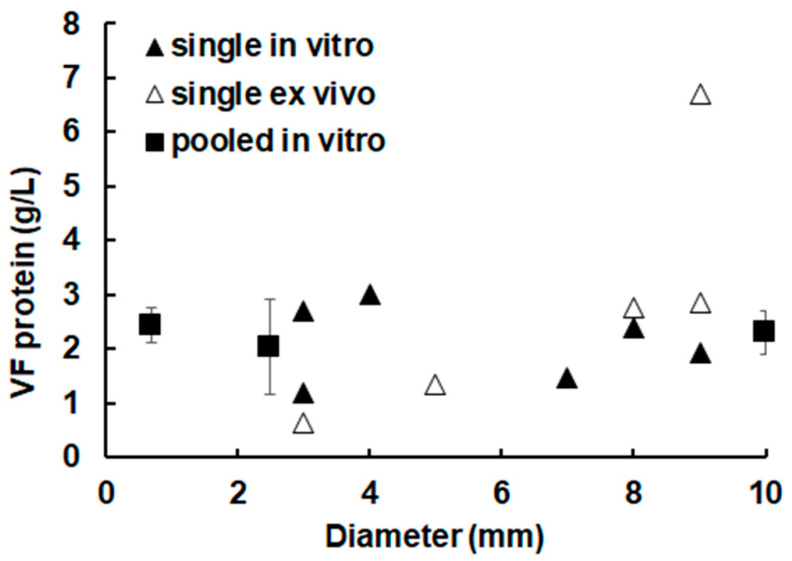
**Protein concentrations in vesicle fluid obtained from single or pooled metacestode vesicles of different sizes.** The protein concentrations were determined using the bichinolic acid (BCA) assay. The in vitro vesicles were incubated in a culture medium (CM) containing 5% fetal bovine serum (FBS). After three days, The vesicles were washed with phosphate buffered saline (PBS), and vesicle fluid (VF) was harvested with a syringe. In the case of pooled vesicles, mean values and standard deviations correspond to three independent pools.

When analyzing the protein concentration in vesicle fluid (VF) of metacestode vesicles of different sizes, it turned out that there was no decrease in protein concentration, i.e., a dilution of proteins, nor an increase in protein concentration, i.e., an accumulation of proteins, as a function of vesicle diameter the concentration fluctuating around 2 g/L in all samples. An exception is, however, observed when regarding vesicles obtained ex vivo, where up to 6 g/L were measured in VF. In these ex vivo vesicles, a tendency to increase protein concentration in larger vesicles could even be observed (Figure 1).

To investigate whether the protein concentration of the VF is correlated with the protein concentration of the medium, metacestode vesicles of three different size classes were incubated for three days in culture medium (CM) containing various amounts of fetal bovine serum. The protein concentrations of the corresponding VFs ranged in all samples between 2.1 and 5.3 g/L, irrespective of the metacestode vesicle size or the protein concentration of the culture medium (Table 1).

### 2.2. Uptake of Host Proteins by Metacestode Vesicles

**Figure 2:**
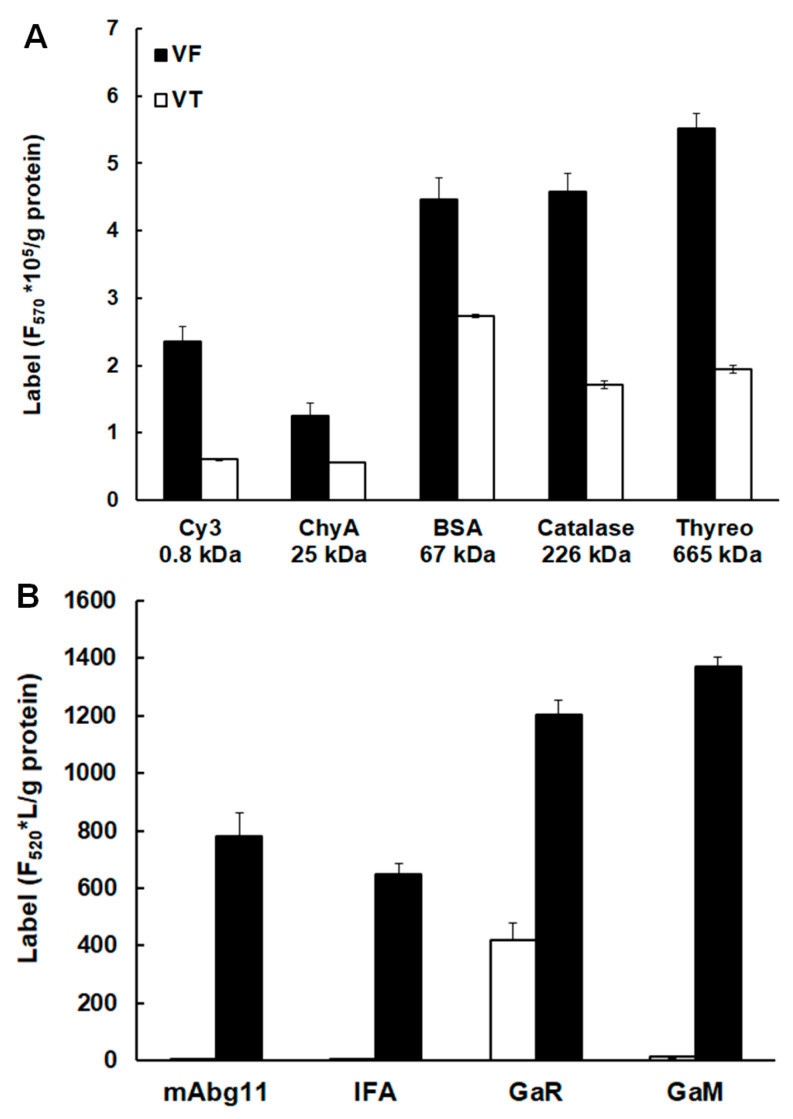
**Uptake of fluorescence-labelled proteins into metacestode vesicles.** (**A**), Intact metacestode vesicles were incubated in PBS buffer containing 200 nM Cy3-labelled chymotrypsin A (Chy A), bovine serum albumin (BSA), catalase or thyreoglobulin (Thyreo). (**B**), Intact metacestode vesicles were incubated in PBS buffer containing FITC-labelled mouse monoclonal antibodies (mAbg11, IFA), goat-anti-rabbit (GaR), or goat-anti-mouse (GaM) IgG (10 mg/L). After 3 h of incubation at 37 °C, the metacestode vesicles were thoroughly washed with PBS, and protein concentrations and label fluorescences of vesicle fluid (VF) and vesicle tissue (VT) were determined. Mean values ± standard deviation are given for three replicates per condition.

When intact metacestode vesicles were incubated in vitro in the presence of various Cy3 or FITC labeled proteins including IgG, the following were identified in VF as well as in VT: labeled thyreoglobulin (665 kDa), catalase (226 kDa), bovine serum albumin (66 kDa), chymotrypsin (25 kDa), as well as free Cy3 (0.8 kDa) (Figure 2A). Moreover, the incubation of metacestode vesicles in the presence of FITC-labelled IgG of both mouse and goat origins resulted in the detection of the label in VF in all cases and in VT in the case of a labeled goat-anti-rabbit IgG (Figure 2B).

### 2.3. Spectrum of Host Proteins Differs Between Compartments

To understand the composition of intermediate host proteins in metacestode vesicles and medium, shotgun LC-MS/MS analyses of these three compartments were performed. The overall results are summarized in Table 2.

Since proteins of both bovine and rat origin are in the medium and since both organisms represent potential hosts of *Echinococcus* spp., these proteins were referred to as “host proteins” in combination in this study.

As expected, the most abundant protein in the conditioned medium was bovine serum albumin, amounting to more than 20% of the total abundance of host proteins, followed by rat serotransferrin and hemiferrin at one magnitude lower abundance (Table 3). The full dataset of medium proteins is presented as Supplemental Table S1. These proteins were still present in VT and VF but at much lower levels. The most abundant proteins showed a different pattern in these compartments as compared to the culture medium. The most abundant host protein in VT was histone H4 (ca. 34% of total proteins), followed by actin and apolipoprotein A-1 (both 13–14%). In VF, the most abundant host protein was alpha2-HS-glycoprotein, with ca. 40% of total host proteins, followed by alpha-antiproteinase or serpin a1 and serum albumin (both around 17%), as shown in Table 3. The full dataset is presented as Supplemental Table S2.

In VF obtained ex vivo (full dataset available as Table S3), serum albumin (here, of mouse origin) was still the most abundant protein followed by hemoglobin, immunoglobulin G (IgG), protein S100-A9 and three histones amongst the eight most abundant proteins. Serum albumin and IgG were also detected in *E. granulosus* hydatid fluid from a human patient [17]. Moreover, serum albumin, IgG, IgM, and hemoglobin were also detected in *E. granulosus* hydatid fluid from a bovine host [18].

**Figure 3:**
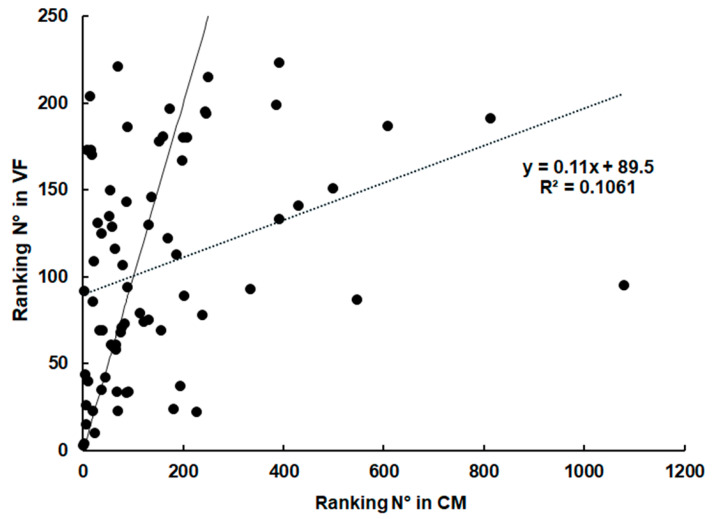
**Ranking of relative abundances of host proteins identified both in culture medium and in vesicle fluid.** The relative abundances were calculated based on the IBAQ values of the respective proteins and ranked from 1 (the most abundant protein within a class) to n (the least abundant protein). The values are listed in Tables S1 and S2. The ideal correlation (r = 1) is presented by a continuous black line. The real correlation is presented as a dotted line. CM, culture medium; VF, vesicle fluid.

The comparison of the eight most abundant proteins suggested an absence of direct correlation between host protein contents in the three compartments: CM, VT, and VF. This view was fostered by subjecting the rankings of protein abundances in the respective compartments to correlation analysis. According to our zero hypothesis, there should be a concentration balance between the compartments. Consequently, the relative abundances should be correlated. To address this, we compared the abundance rankings of proteins found both in CM and VF. As shown in Figure 3, there is only a weak correlation (r = 0.33) between the rankings in both compartments.

**Figure 4:**
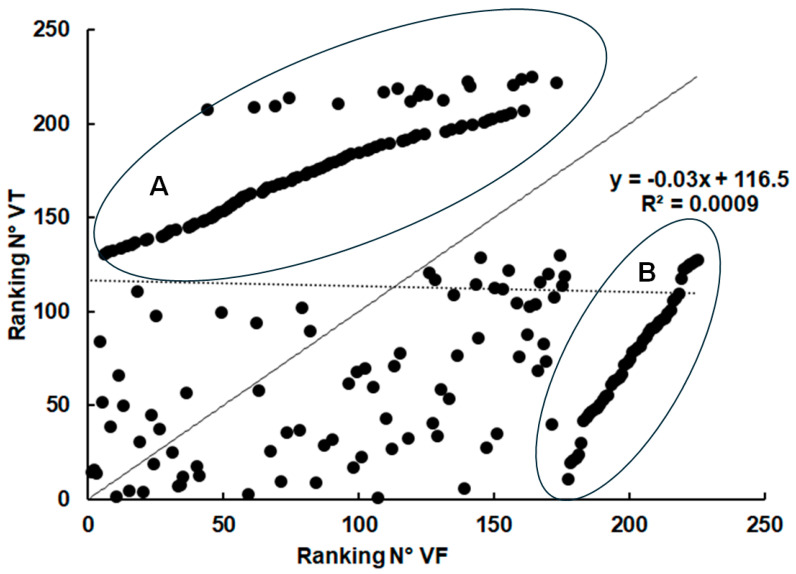
**Ranking of relative abundances of proteins identified both in vesicle tissue and in vesicle fluid.** The relative abundances were calculated based on the IBAQ values of the respective proteins and ranked from 1 (the most abundant protein within a class) to n (the least abundant protein). The values are listed in Table S2. The ideal correlation (r = 1) is presented by a continuous black line. The real correlation is presented as a dotted line. A, group of proteins overrepresented in vesicle fluid (VF). B, group of proteins overrepresented in vesicle tissue (VT).

When analyzing the abundance rankings of host proteins in VF and VT, even a complete absence of correlation (r = 0.03) was noted. However, two distinct subsets of host proteins could be identified in these compartments, one overrepresented in VF and at or below the detection limit in VT (Figure 4, group A), one overrepresented in VT and below the detection limit in VF (Figure 4, group B).

**Figure 5:**
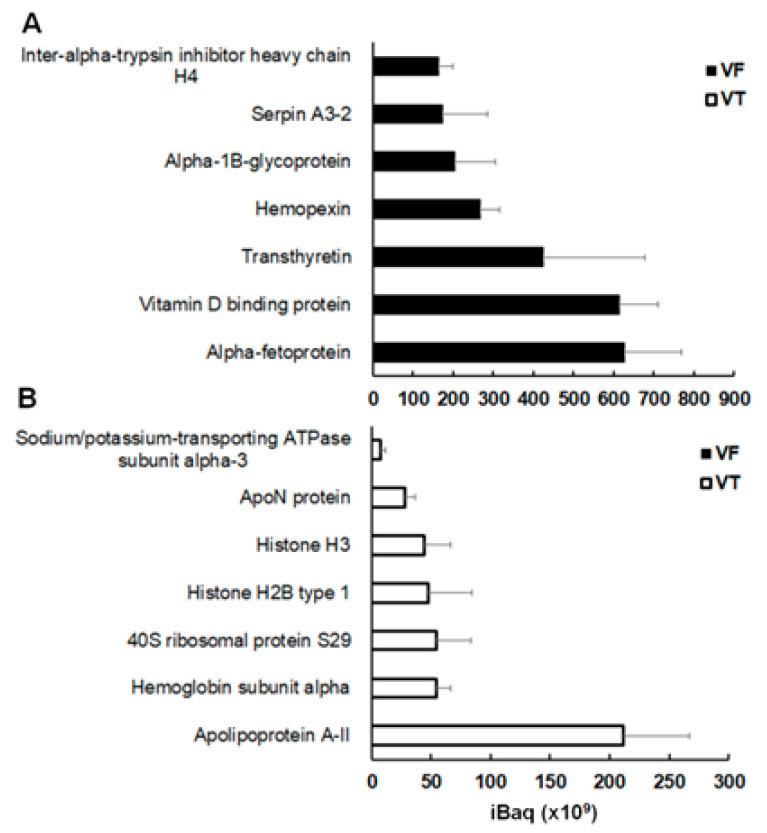
The seven most abundant proteins of group (**A**) (overrepresented in vesicle fluid) and group (**B**) (proteins overrepresented in vesicle tissue) as depicted in Figure 3 and listed in Table S2. Mean IBaq values ± standard deviations are given for five biological replicates. VF, vesicle fluid; VT, vesicle tissue.

In VF, the three most abundant group A proteins are alpha-fetoprotein, vitamin D binding protein, and transthyretin, with mean IBaq values around 5 × 10^11^ (Figure 5A). These proteins were below the detection limit in VT. Furthermore, amongst the seven most abundant group A proteins were two serine protease inhibitors, namely serpinA3-2 and inter-alpha-trypsin inhibitor heavy chain H4. In total, those six other serine protease inhibitors were in this group of proteins (Table S2).

Conversely, group B, representing proteins overrepresented in VT and almost absent in VF, was dominated by apolipoprotein A-II (IBaq 2 × 10^11^), followed by four other proteins with nearly one-quarter of this abundance. These four proteins were hemoglobin subunit alpha, a ribosomal protein, and two histones (Figure 5B).

## 3. Discussion

In this study, we have shown that host proteins pass from the surrounding medium into *E. multilocularis* vesicle fluid (VF) irrespective of their size or charge, thereby confirming previous results [18,20]. Alone or combined with endogenous proteins, these host proteins may have various functions. First, the proteins and other polymers within the vesicle adsorb water, thereby increasing the colloid-osmotic or oncotic pressure, which—together with the osmotic pressure (proportional to the molarity of solutes only)—causes an influx of water maintaining the vesicle turgid and with an open lumen within the surrounding tissue (see, e.g., [26] for general considerations concerning water potentials). For instance, serum albumin, the most abundant protein in host serum [27], as well as an abundant host protein in vesicles, has a water binding capacity of ca. 170 mg of tightly sorbed water per gram of albumin [28]. This space is a prerequisite for protoscolex formation within the metacestode. After ingestion by a definitive host, protoscoleces transform into adult worms, thereby completing the developmental cycle. Thus, anything promoting protoscolex development contributes ipso facto to Darwinian fitness. The metacestode thus compares to a vertebrate amnion filled with amniotic fluid. The protein concentration of the metacestode fluid remains constant during growth, even in the presence of high protein concentrations in the culture medium (CM). This is different from the situation, e.g., in human amniotic fluid, where the protein concentration first increases and then markedly decreases towards the term of gestation at the benefit of the embryo [29,30]. The increase in volume of amniotic fluid is thus rather due to an influx of electrolytes increasing the osmotic pressure [31] than to the accumulation of proteins increasing the oncotic pressure.

A second function of host proteins within the metacestode may reside in providing nutrients, thereby fueling growth and differentiation into protoscoleces, both aspects contributing to Darwinian fitness once again. The host proteins may serve as food by directly providing (essential) amino acids to the growing and differentiating metacestode after cleavage by endo- and exopeptidases. In fact, metacestodes contain cathepsin-like cysteine peptidases [32] acting on a variety of host proteins, including serum albumin and immunoglobulins [33], and not only on preselected peptides of specific interest, such as, e.g., eotaxin [34]. Moreover, metalloproteinases have been identified in *E. granulosus* [35] and leucine aminopeptidases in *E. granulosus* [36] and *E. multilocularis* [37] metacestodes. Conversely, the presence of serine protease inhibitors called serpins in metacestodes [38,39] suggests a preference for the above-mentioned peptidases over serine proteases for host protein degradation. This view is fostered by our own observation that serpins and other serine protease inhibitors of host origin are enriched in the VF over vesicle tissue (VT).

Furthermore, host proteins taken up into metacestodes may transport micromolecular nutrients. The most predominant host protein in VF, apha-2-HS-glycoprotein, interacting with fetuin, another abundant host protein, forms calciprotein particles, preventing the unwanted precipitation of calcium phosphate. These particles may contribute to the import of calcium into and storage within the vesicle [40,41]. Besides its multiple other properties [42], serum albumin is a notorious fatty acid-binding protein [43] and, therefore, in all likelihood, contributes to fueling the metacestode with nutrients. Other, more specific nutrient-binding proteins found in metacestodes are the apolipoproteins AI and AII, both enriched in VT over VF. Both proteins are components of high-density lipoproteins [44] and thus are involved in the transport of cholesterol from the periphery to the liver [45,46]. The uptake of cholesterol-loaded lipoproteins into the metacestode could contribute to fueling proliferating cells in the germinal layer. Serotransferrin, one of the more abundant proteins found in VF as well as in CM, is the predominant iron transport protein in human plasma [47]. Since a constant supply of iron is essential for proliferating germinal layer cells as well as for host cells, the serotransferrin within the VF can be considered as a reservoir maintaining this essential iron homeostasis. Moreover, host-derived hormones and vitamin transporters are accumulated in VF, in particular transthyretin, the major thyroid hormone binding (and therefore iodine carrier) protein [48], and a vitamin D binding protein, the primary transporter of the steroid vitamin D [49].

A third major host protein group of interest identified in metacestode vesicles—although not abundant in the CM—is histones. Principal constituents of chromatin under physiological conditions, histones may be released to the medium after cellular damage and act as mediators of innate immunity by stimulating cytotoxicity, cells of the immune system, and coagulation [50]. Consequently, interference with extracellular histones contributes to dimming inflammatory reactions [51]. Another inflammation-related protein scavenged by metacestodes is the protein S100-A9. This protein is considered an alarmin [52] promoting inflammation via nuclear factor-kappaB activation [53,54].

Therefore, it is not surprising that *E. multilocularis*, a master in avoidance of innate and acquired immune responses (see, e.g., [55,56] for review), avidly scavenges inflammation-stimulating proteins from the medium. Scavenging histones means hitting two birds with one stone, namely dimming host inflammatory reactions and fueling cell proliferation and, ultimately, differentiation into protoscoleces with the essential amino acid lysine, a major constituent of histones, and by stimulating the uptake of serum albumin [57].

This leads to our initial question of whether the uptake of host proteins into the metacestode occurs unspecifically. Labeled proteins with diverse physicochemical properties are taken up into metacestode vesicles under artificial conditions, and in vitro and in vivo, the most abundant proteins in the medium, serum albumin in particular, are also amongst the most abundant proteins in metacestode VF. Nevertheless, comparing the respective amounts of these proteins in CM, VT, and VF, we do not see a strict correlation, thereby falsifying our zero hypothesis that this uptake occurs unspecifically. How can this preference be explained? As shown by a biotin labeling study, the uptake of supercharged proteins from the extracellular medium into endosomes is favored over, e.g., cell-penetrating peptides [58]. The fact that the endophilin homolog protein p29 is amongst the most abundant *E. multilocularis* proteins in the VT [25] suggests a major role of endocytosis in protein uptake from the medium into germinal layer cells. Consequently, the host proteins have to cross the laminated layer, where they may be passively retained (and stored) as a function of their charges (e.g., positively charged histones by carboxyl groups of the extracellular matrix). Unfortunately, besides its role as a potential vaccine documented in numerous articles (see, e.g., [59,60,61]), p29 has not been characterized so far with respect to its biological function.

**Figure 6:**
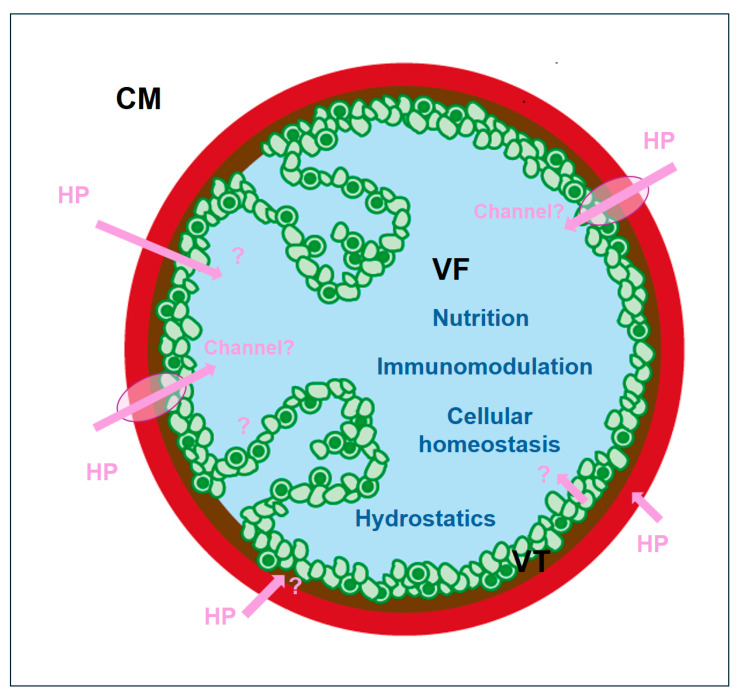
Schematic representation of potential import routes and roles of host proteins within *E. multilocularis* metacestode vesicles. CM, culture medium; HP, host protein (pink); VF, vesicle fluid (light blue); VT, vesicle tissue, including GL, germinal layer (green); LL, laminated layer (red); T, tegument (brown). Invaginations from the GL represent the formation of brood capsules. Figure adapted from reference [63].

Moreover, host proteins may cross the laminated and germinal layers by alpha-helical channels, favoring the passage of proteins over small molecules [62]. At specific sites within the metacestode tissue, such hypothetical channels may translocate proteins directly from the medium into the metacestode lumen. Moreover, *E. multilocularis* and other helminths may possess genuine protein uptake mechanisms unknown, so far, in other organisms. Figure 6 summarizes the roles and potential uptake mechanisms of host proteins in *Echinococcus* metacestodes.

So far, the uptake mechanisms mentioned above are hypothetical since appropriate investigation tools are lacking in *Echinococcus*, which is the opposite of the situation in model mammalian cell lines or yeast. A future strategy could be to express candidate *Echinococcus* genes in these model systems and to look for gain-of-function phenotypes (e.g., increased uptake of one or several host proteins investigated in this study). One should, however, be aware that the transformation procedure by itself may cause artifacts and include appropriate controls in this type of experiment (see, e.g., [64] for review). For knock-out/knock-in studies, the controls comprise irrespectively of the method chosen: (i) verification of absence/presence of the protein of interest by proteomics; (ii) phenotypical comparison of the transformed strain of interest, a strain transformed with an irrelevant protein, and an untransformed wild type, including the analysis of differentially expressed proteomes in all strains.

Finally, we raise the question of whether our results are of importance for the improvement of AE and CE therapies. With all due respect to scientific progress, we must be aware that there is only little investment in drugs for neglected diseases. Targeted drug delivery, i.e., using selected host proteins loaded with a suitable drug as a kind of guided missile, may sound—prima vista—seducing from an academic point of view, but it is closer to Science Fiction than to science as a part of realistic treatment. Overall, the current treatment of echinococcosis comprising surgery and/or chemotherapy based on benzimidazoles may be improved by an appropriate diet, e.g., in the case of anti-cancer therapy [65]. Our results presented here, as well as previously published findings, [24,66] suggest that proteins, together with free amino acids and lipids, play an important role in fueling the proliferation and differentiation of metacestodes. If this is the case, a diet with reduced input of amino acids, proteins, and lipids in combination with current treatments might enhance the therapeutic success.

## 4. Materials and Methods

### 4.1. Chemicals

All chemicals used were purchased from Sigma (St. Louis, MO, USA). Cell culture media and fetal bovine serum (FBS) were from Bioswisstec (Schaffhausen, Switzerland).

### 4.2. In Vitro Culture of E. multilocularis Metacestodes

Metacestode in vitro growth medium was prepared by preconditioning Dulbecco’s Modified Essential Medium (DMEM) with Reuber rat hepatoma cells (RH) purchased from ATCC (Molsheim, France) as described in Ritler et al. [24]. The studies presented here were performed using *E. multilocularis* isolate H95 metacestode vesicles grown in vitro for 12 weeks supplemented with 5% fetal bovine serum (FBS). For the determination of protein concentration at various FBS concentrations, vesicles of different sizes (<2 mm to 10 mm) were incubated in 6-well plates containing 5 mL medium with FBS ranging from 5% to 50% for 3 days. Proteomic analyses were performed using vesicles with a diameter between 2 and 4 mm. Protein concentrations of VF harvested by syringe were determined using metacestode vesicles with diameters between 0.5 and 10 mm by bichinolinic acid assay (BCA assay; Thermo Fisher Scientific, Waltham, MA, USA). Metacestode vesicle integrity was visually confirmed during the experiment and before the collection of samples.

### 4.3. Parasite Maintenance and Ex Vivo Sample Preparation

Animals for parasite maintenance were purchased from Charles River Laboratories (Sulzheim, Germany) and used for parasite maintenance after 2 weeks of acclimatization. BALB/c mice were maintained in a 12 h light/dark cycle at a controlled temperature of 21–23 °C and a relative humidity of 45–55%. Food and water were provided *ad libitum*. All animals were treated in compliance with the Swiss Federal Protection of Animals Act (TSchV, SR455) and were approved by the Animal Welfare Committee of the canton of Bern under the license numbers BE2/22 and BE30/19. *E. multilocularis* metacestodes (isolate H95) were grown in intraperitoneally infected mice for approximately 4 months. The mice were euthanized with CO_2_ after 3–4 months of parasite growth. VF of five independently infected animals was aseptically removed by syringe and stored at −80 °C until further analysis.

### 4.4. Fluorescence Labeling of Marker Protein

To perform uptake studies with defined, labeled xenoproteins, purified chymotrypsinogen A (25 kDa), bovine serum albumin (67 kDa), catalase (226 kDa), and thyreoglobulin (665 kDa) from a marker kit for size exclusion chromatography (Thermo Fisher Scientific, Waltham, MA, USA) were labeled with Cy3 (0.8 kDa) using a commercial kit and the amount of incorporated label was quantified according to the manufacturer’s instructions (Promega, Madison, WI, USA). Moreover, FITC labeled mouse monoclonal antibodies g11 and IFA, as well as FITC, labeled goat-anti-mouse and goat-anti-rabbit IgG ((Thermo Fisher Scientific, Waltham, MA, USA), was used for uptake studies.

### 4.5. Proteomics

For subsequent analysis by LC-MS/MS, the metacestode CM of the above-described setup was collected, centrifuged for 10 min at 500× *g* and 4 °C, and immediately stored at −80 °C. The metacestode vesicles were washed three times in 4 °C cold NaCl (0.9%), broken with a 1 mL pipette tip, and centrifuged at 9000× *g* for 20 min at 4 °C. The supernatant was removed and centrifuged again at 12,000× *g* for 20 min, 4 °C yielding VF. The remaining metacestode tissue was washed with NaCl (0.9%, 4 °C) and centrifuged as described above, yielding VT. Both preparations were stored at −80 °C for subsequent protein analysis by LC-MS/MS performed as described in Müller et al. [25]. The original MS/MS data are available on Pride ProteomeXchange with access number PXD040274

(https://proteomecentral.proteomexchange.org/cgi/GetDataset?ID=PXD040274, accessed on 15 January 2025). The data were analyzed using the uniport database (www.uniprot.ch; accessed on 15 January 2025).

## Figures and Tables

**Table 1:** **The protein concentration of vesicle fluid is not correlated with the protein concentration of the medium.** *E. multilocularis* metacestode vesicles were divided into the three size classes S (< 2 mm in diameter), M (4–6 mm), and L (>8 mm) and incubated in 6-well-plates filled with 5 mL/well culture medium (CM) containing various amounts of FBS. After three days, vesicles were washed with PBS, the vesicle fluid (VF) was harvested with a syringe, and the protein concentrations of VF and CM were determined using the BCA assay. Mean values and standard deviations correspond to three independent pools.

FBS (%)	CM Protein (g/L)	Size Class	VF Protein (g/L)
		S	4.4 ± 0.7
5	2.6 ± 0.2	M	5.2 ± 0.8
		L	5.2 ± 0.8
		S	3.1 ± 0.5
10	3.8 ± 0.4	M	2.9 ± 0.7
		L	5.3 ± 2.2
		S	3.8 ± 0.1
20	5.5 ± 0.5	M	5.1 ± 0.6
		L	4.3 ± 1.1
		S	2.1 ± 0.2
50	11.5 ± 1.0	M	4.4 ± 1.7
		L	3.4 ± 1.0

**Table 2:** Summary of host protein quantification data in *Echinococcus multilocularis* metacestodes in vitro and ex vivo, and in culture medium. Analyses were performed by LC-MS/MS on five biological replicates within each dataset.

Samples Analyzed	Unique Peptides	Non Redundant Proteins	Full Dataset
Culture medium (CM)	12372	1170	Table S1
Vesicle fluid and tissue (VF and VT)	1932	225	Table S2
Vesicle fluid (VF) ex vivo	14730	1120	Table S3

**Table 3:** List of the eight most abundant host proteins in medium, vesicle tissue, and vesicle fluid of in vitro cultured metacestode vesicles and of vesicle fluid obtained ex vivo. Relative abundances calculated from peptide match score summation (PMSS) values (CM) or intensity-based absolute quantification (IBaq) values (VT and VF) are given as percentages of total host proteins identified in the respective compartments. Mean values ± standard deviations are given for five biological replicates.

Compartment	Protein	Accession	Origin	Rel. abu. (%)
**Culture medium (CM)**				
	Serum albumin	P02769	bovine	21.4 ± 4.1
	Serotransferrin	P12346	rat	2.0 ± 0.4
	Hemiferrin	Q64599	rat	1.9 ± 0.9
	Alpha-2-HS-glycoprotein	P24090	rat	1.8 ± 0.3
	Serum albumin	A0A0G2JSH5	rat	1.7 ± 0.3
	Heat shock protein HSP 90-beta	P34058	rat	1.6 ± 0.3
	Complement component C2	Q8CIP8	rat	1.5 ± 0.3
	Keratin, type II cytoskeletal	Q6IMF3	rat	1.3 ± 0.2
**Vesicle tissue (VT)**				
	Histone H4	G3X807	bovine	34.4 ± 6.9
	Actin, alpha cardiac muscle 1	Q3ZC07	bovine	14.0 ± 2.2
	Apolipoprotein A-I	V6F9A2	bovine	12.8 ± 1.5
	Peroxiredoxin-4	Q9Z0V5	rat	7.5 ± 0.8
	Heat shock protein HSP 90-beta	A0A0G2K793	rat	5.1 ± 0.3
	Hemoglobin fetal subunit beta	P02081	bovine	4.0 ± 1.0
	Tubulin beta-4B chain	Q6P9T8	rat	2.8 ± 0.1
	Triosephosphate isomerase	Q6SA19	rat	2.6 ± 0.3
**Vesicle fluid (VF)**				
	Alpha-2-HS-glycoprotein	B0JYN6	bovine	40.6 ± 5.7
	Alpha-1-antiproteinase	P34955	bovine	17.0 ± 5.5
	Serum albumin	P02769	bovine	16.2 ± 9.4
	Serotransferrin	Q29443	bovine	5.5 ± 2.8
	Fetuin-B	Q58D62	bovine	3.7 ± 0.8
	Alpha-fetoprotein	Q3SZ57	bovine	1.7 ± 0.4
	Vitamin D binding protein	I7CT57	bovine	1.6 ± 0.3
	Albumin	B0JYQ0	bovine	1.3 ± 0.8
**Vesicle fluid (VF) ex vivo**				
	Serum albumin	P07724	mouse	6.2 ± 1.4
	Hemoglobin subunit beta-1	P02088	mouse	4.6 ± 3.1
	Histone H2B type 1-C/E/G	Q6ZWY9	mouse	4.0 ± 2.8
	Hemoglobin subunit alpha	P01942	mouse	3.7 ± 2.0
	Immunoglobulin gamma-1 chain C region	P01868	mouse	3.6 ± 1.3
	Protein S100-A9	P31725	mouse	3.6 ± 2.4
	Histone H2A type 1-H	C0HKE9	mouse	3.3 ± 0.4
	Histone H3.2	P84228	mouse	3.0 ± 1.2

## Data Availability

The original MS/MS data are available on Pride ProteomeXchange with access number PXD040274.

## Supplementary Materials

The following supporting information can be downloaded at https://www.mdpi.com/article/10.3390/ijms26073266/s1.
